# Pyroptosis-Related Signatures for Predicting Prognosis in Breast Cancer

**DOI:** 10.3389/fsurg.2022.788437

**Published:** 2022-02-08

**Authors:** Tong Ren, Xuhui Guo, Jingyang Zhang, Zhenzhen Liu

**Affiliations:** Department of Breast Disease, Henan Breast Cancer Center, The Affiliated Cancer Hospital of Zhengzhou University and Henan Cancer Hospital, Zhengzhou, China

**Keywords:** breast cancer, women in oncology, surgical oncology, pyroptosis, LASSO Cox analysis

## Abstract

**Background:**

Female breast cancer (BC) has become the most common cancer in the world, and its mortality was considerably higher in transitioning vs. transitioned countries. Pyroptosis, an inflammation-dependent programmed cell death mediated by inflammasomes, has been observed in human colorectal tumors and gliomas. However, the characteristics of pyrolysis-related genes and their influence and mechanism on the tumorigenesis and progress of BC were unknown.

**Methods:**

Based on the global public database, we used comprehensive bioinformatics analysis to systematically analyze the expression of pyroptosis-related genes in BC and their relationship in tumor progression. In addition, BC patients were divided into two groups, and the clinical features and outcomes could be better predicted by the consistent clustering of pyroptosis-related genes. Least absolute shrinkage and selection operator (LASSO) Cox regression analysis was used to establish a risk score. Then, we further explored the prognostic value and clinical features of pyroptosis genes. Finally, we used the Human Protein Atlas (HPA) platform to identify the expression at protein levels of the key genes.

**Results:**

We confirmed that the expression of pyroptosis-related genes was different in BC and normal breast tissues. A high frequency of somatic mutations occurred in BC. In addition, 33 pyroptosis-related proteins interacted frequently. Based on univariate analysis and the LASSO Cox model, five pyroptosis-related genes [including GADMA, interleukin-6 (IL-6), NLR pyrin domain-containing protein 6 (NLRP6), caspase-1 (CASP1), and caspase-9 (CASP9)], were obtained to calculate a risk score. The risk score was identified as an independent risk factor for the prognosis of BC and might play an auxiliary role in clinical classification. The HPA platform confirmed that the expression trends of the key genes were consistent with our previous studies.

**Conclusion:**

Pyroptosis had an important effect on the progression of BC. And the pyroptosis-related genes could be used as new prognostic biomarkers and therapeutic targets for BC.

## Introduction

Breast cancer (BC) has become the first malignant tumor with morbidity and mortality among women in the world (1), which seriously endangers women's life and health. The incidence of BC has increased significantly, and its mortality rate has also shown an upward trend in fluctuation view of the existing research results. The incidence of BC has increased slightly by 0.3% per year since 2012 (2). Therefore, it is urgent and critical to developing powerful prognostic predictors and new therapeutic targets to enhance the prognosis assessment and treatment level of BC.

Pyroptosis, also known as cell inflammatory necrosis, was a new form of programmed cell death closely related to inflammation (3). When the cell pyroptosis occurred, the cell swelled. Before the cell ruptured, a protrusion was formed on the cell, and then a pore was formed in the cell membrane, which made the cell membrane lose its integrity, released the cell content, and triggered an inflammatory response (4). As the morphology changed, the nucleus shrank and DNA broke (5). The pyroptosis process involved many inflammation-related molecules such as gasdermin (GSDM) protein family, NOD-like receptors (NLRs), interleukin (IL) series molecules, caspase molecules, etc. The caspase family belonged to cysteine proteases, which were key enzymes that cause cell apoptosis (6). Once activated by signal pathways, they could degrade intracellular proteins and lead to cell death. Interleukins and related cytokines were the communication means of innate and adaptive immune cells and non-immune cells and tissues (7). Interleukins affected the occurrence, development, and control of cancer. NOD-like receptors were a subgroup of cytoplasmic host pattern recognition receptors (8). NOD-like receptors was involved in the formation of the inflammasome polyprotein complex, which induced the release of interleukin-1β (IL-1β) and interleukin-18 (IL-18) leading to a pro-inflammatory response (9). Gasdermins were a family of intracellular proteins that executed pyroptosis (10, 11). Caspase-1 (CASP1) and caspase-11/4/5 induced pyroptosis through cleavage Gasdermin D (GSDMD). Gasdermin D released its N-terminal domain after being cleaved by CASP1 or caspase-11/4/5 (12). It had the activity of binding membrane lipids and punching holes in the cell membrane, leading to changes in cell osmotic pressure and swelling until the final cell membrane ruptured (13). However, there were few studies on its specific functions in BC. We researched the role of pyroptosis-related genes in BC.

In this study, we firstly conducted a systematic study on the TCGA database and the GSE45628 microarray database to evaluate the difference in pyroptosis-related genes expression between non-specific invasive BC tissues and the paracarcinoma tissues. Then we discussed whether these genes had an impact on the prognosis and constructed a prognostic evaluation model through the least absolute shrinkage and selection operator (LASSO) analysis to help judge the progress and prognosis of clinical patients after standard treatment, including the NLR pyrin domain-containing protein 6 (NLRP6), interleukin-6 (IL-6), CASP1, caspase-9 (CASP9), and GSDMD. According to the relative coefficients, these key genes played a vital part in suppressing cancer in the effect of pyroptosis on BC. Finally, we further studied and validate the expression of the key genes in the Human Protein Atlas (HPA) platform.

## Materials and Methods

### Public Databases

We selected the data set according to the following criteria: Inclusion criteria: datasets involving human BC and expression profiling by the array. Exclusion criteria: datasets with a sample size smaller than 10 and datasets without follow-up or metastasis information. We obtained public RNA sequencing (RNA-seq) data and corresponding clinical features including 1,213 BC patients and 130 normal people. The training set came from The Cancer Genome Atlas (TCGA; https://tcga-data.nci.nih.gov/tcga/, *n* = 1,222, 1,109 BC samples and 113 normal breast samples), the validation set was a separate Gene Expression Omnibus (GEO) microarray dataset (GSE42568, *n* = 121, 17 normal breast samples, 104 BC samples) (14). The above data were all publicly accessible resources, so ethical approval was not required. The characteristics of BC patients and normal people were shown in Supplementary Table 1. The human protein–protein interactions (PPI) were compiled from the Human Integrated Protein–Protein Interaction rEference (HIPPIE) database (http://cbdm.mdc-berlin.de/tools/hippie/hippie_current.txt) (15).

### Consensus Clustering

From the TCGA database, 33 previously reported expressions related to pyroptosis were extracted. The BC Patients without follow-up information were deleted. After that, with the help of the “Consensus Cluster Plus (16)” package, the above steps and 1,000 times repetitions for guaranteeing the stability of clustering. The optimal number of clusters were determined according to the consensus clustering algorithm. At last, two clusters were determined according to the expression profile of pyroptosis-related genes in BC patients.

### Pathway Enrichment Analysis

Using Spearman's analysis and the String online tool (https://string-db.org/), the correlation and interaction of 33 pyroptosis-related genes were evaluated. We used Metascape (http://metascape.org/) to complete the assessment of the biological behavior of different clusters. Gene Set Variation Analysis (GSVA) was used to derive the landmark pathways described in the molecular feature database and conducted pathway analysis to determine the potential mechanisms involved in the molecular cluster of pyroptosis. After that, the Gene Ontology (GO) and Kyoto Encyclopedia of Genes and Genomes (KEGG) pathway analysis were performed with the help of Gene Set Enrichment Analysis (GSEA), using cluster Profiler, an R/Bioconductor package.

### Composing Risk Score and Verification

In order to construct powerful prognostic characteristics, all molecules related to pyroptosis obtained by non-factor analysis were included in the LASSO Cox regression model. We incorporated the genes obtained after the initial screening of univariate analysis into the Lasso penalty Cox regression, and the genes related to the prognosis of BC were further analyzed to identify potential prognostic genes. Then, they derived the most suitable lambda value by a five-fold validation through the R package “glmnet” to minimize the average cross-validation error (30). After screening, it was finally determined to use five key genes and their coefficients to construct a prognostic model. The formula format of the risk score was as follows:


$$\text{Risk score = }\sum_{i\;=\;1}^{n}Coefi^{*}xi$$

where *Cofi* was the coefficient, *xi* was the pyroptosis-related genes, and the mRNA expression value of the genes. This formula would calculate the risk score of all patients in our study.

### Immunohistochemical Data of Partial Pyroptosis-Related Genes Expression

We used the data provided by the HPA (https://www.proteinatlas.org/) to examine immunohistochemical (IHC) staining of GADMA, IL-6, NLRP6, CASP1, and CASP9 in normal breast tissue and non-specific invasive BC cases. Based on a comprehensive assessment of the staining intensity (negative, weak, medium, or strong) and the proportion of stained cells (<25, 25–75, or >75%), manually scored protein expression in the database.

### Statistical Analysis

Unless otherwise stated, all statistical analyses were conducted by R software (version 3.5.1). We got help from the Student's *t*-test (unpaired, two-tailed) to compare the difference between two independent groups. The correlation between pyroptosis-related genes, risk scores, and clinical characteristics was calculated by the Chi-square test. The Kaplan-Meier analysis of Disease-Free Survival (DFS) and Overall Survival (OS) was performed using a log-rank test according to the best cut-off value. We subsequently performed univariate and multivariate Cox regression analysis to determine the relationship between different variables and clinical outcomes. The *P*-values were corrected for multiple comparisons via the Benjamini and Hochberg (BH). The *P*-value < 0.05 was considered statistically significant.

## Results

### Differential Expression of Pyroptosis-Related Genes in Breast Cancer and Paracancer Tissues

**Figure 1:**
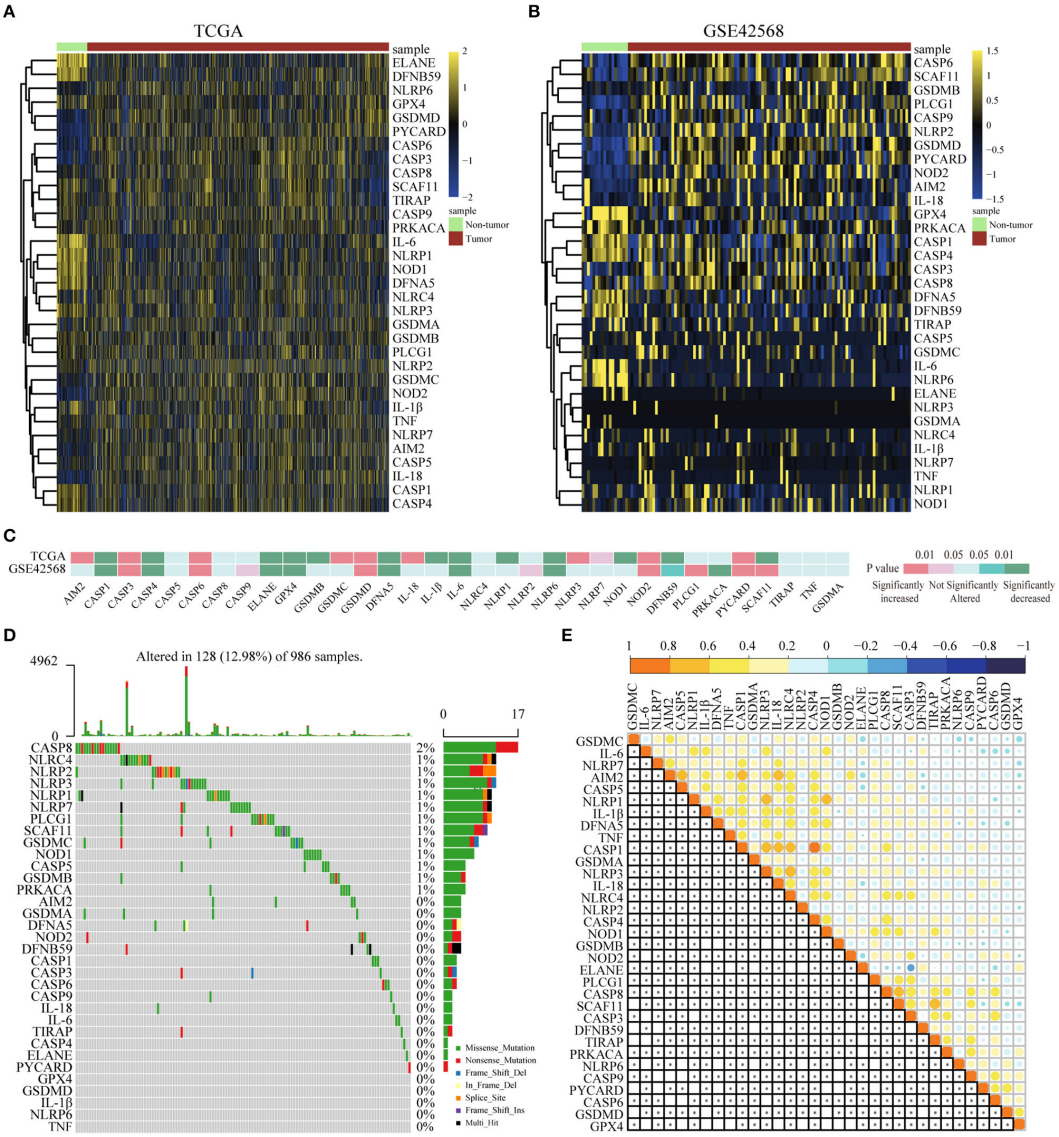
Differential expression of pyroptosis-related genes in BC and paracancer tissues. **(A)** The expression of pyroptosis-related genes in BC and paracancer tissues in the TCGA database. **(B)** The expression of pyroptosis-related genes in BC and paracancer tissues in the GSE45628 database. **(C)** The mRNA expression status of pyroptosis-related genes in BC. **(D)** The somatic mutation frequency in pyroptosis-related genes of the TCGA database. **(E)** Pearson's correlation analysis of the expression of 33 pyroptosis-related genes in BC. BC, breast cancer.

For the purpose of exploring the important biological functions of pyroptosis-related genes in BC occurrence and tumor progression, based on GEO and TCGA, 33 pyroptosis-related genes were compared between BC tissues and paracarcinoma tissues in the mRNA and protein levels. First of all, we found that the expression of 33 genes was disordered in BC tissues and paracarinoma tissues in the TCGA dataset (Figure 1A), which were verified in GSE42568 (Figure 1B). Further analysis of the specific differences indicated that the expression level of five genes (CASP3, CASP6, GSDMD, NOD2, PYCARD) was prominently increased in BC tissues. Another eight genes were observably lower expressed in BC tissues (Figure 1C). In addition, we tried to further explain the reasons for the alterations in the expression of pyroptosis-related genes by the somatic mutations of the differential genes. The analysis results showed that 128 out of 986 samples had mutations, which the mutation rate was 12.98%. The rate was relatively high, and somatic mutations of differential genes were common in BC patients (Figure 1D). Subsequently, we utilized Pearson correlation analysis to verify the relationship between the expression profiles of 33 pyroptosis-related factors in the TCGA database (Figure 1E). As shown in the figure, most of the pyroptosis-related genes were positively correlated, and the expression of AIM2, CASP5, CASP1, CASP4, IL-18, NLRP1, NLRP3, and SCAF11 showed strong correlations in BC. To sum up, the above results showed that there was a large genomic and expression variation of pyroptosis-related genes between BC tissues and paracarinoma tissues. Furthermore, the effect of pyrolysis-related genes on prognosis in BC was shown in Supplementary Figures 1, 2.

### Interaction and Unsupervised Consensus Analysis of 33 Pyroptosis-Related Genes

To achieve the aim of identifying the relation between pyroptosis-related genes and clinicopathological characteristics in BC, we systematically studied the functions, interactions, and correlations of pyroptosis-related genes. It could be seen from the figure that there was a strong relationship in the PPI network (Supplementary Figure 3A). Univariate analysis showed that IL-18 is a potential protective factor, and TIRAP was a potential risk factor for OS (Supplementary Figure 3B). In addition, CASP9 was a potential protective factor for DFS in pyroptosis (Supplementary Figure 3C).

**Figure 2:**
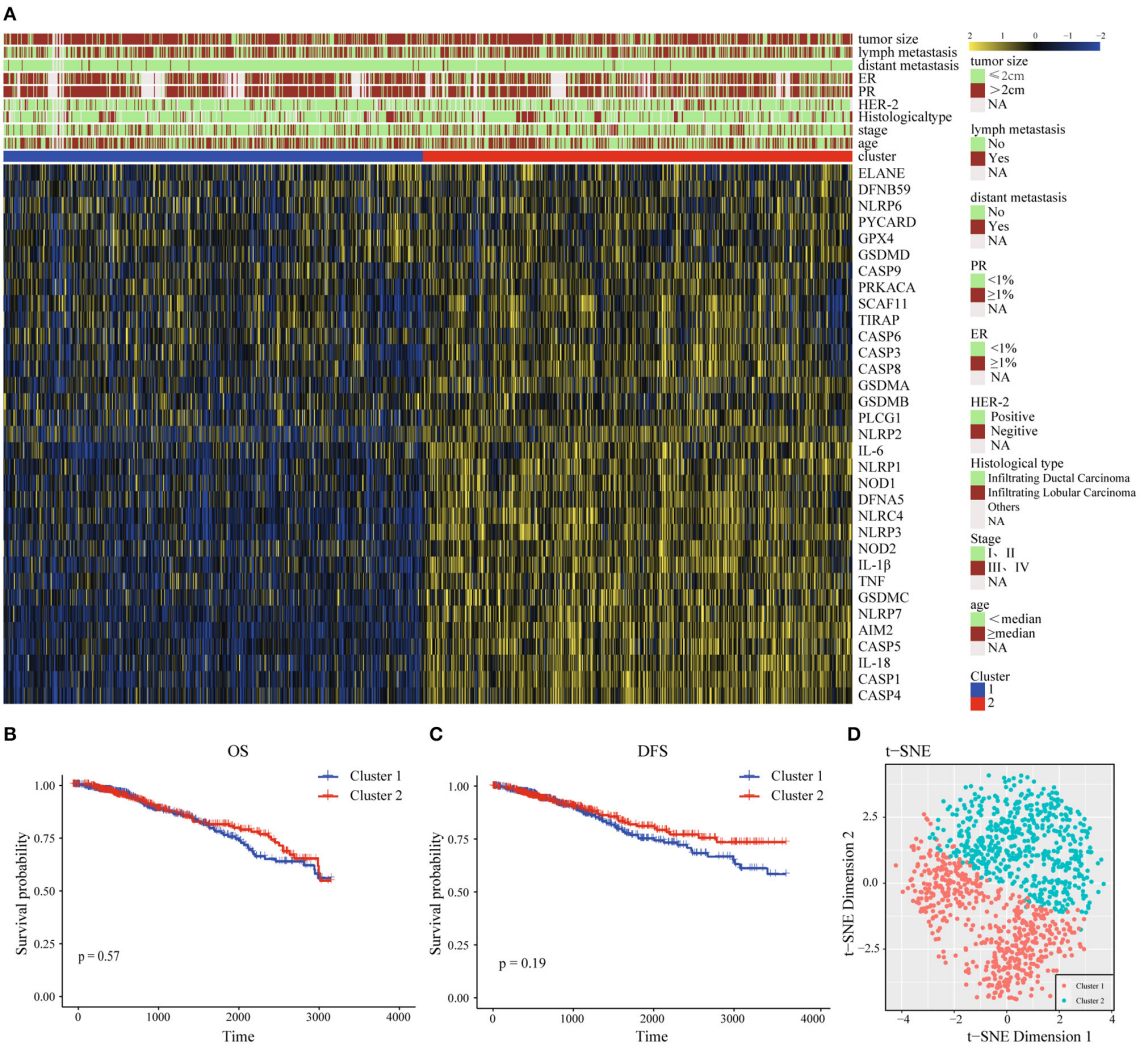
Interaction and unsupervised consensus analysis of these pyroptosis-related-genes. **(A)** The relationship between the clinical features and the expression levels of pyroptosis-related genes in the two clusters of BC. **(B)** Overall Survival analysis of two clusters of BC patients. **(C)** Disease-Free Survival analysis of two clusters of BC patients. **(D)** t-SNE plots of TCGA-BC RNA-sequence profiles for the two clusters. t-SNE, t-distributed stochastic neighbor embedding; DFS, Disease-Free Survival; OS, Overall Survival; HER-2, Human Epidermal Growth Factor Receptor 2; PR, progesterone receptor; ER, estrogen receptor.

For the purpose of exploring the relationship between 33 pyroptosis-related differently genes (DEGs) and BC subtypes, we performed an unsupervised and consistent cluster analysis based on the TCGA cohort, by changing the clustering variable (*k*) from 2 to 6 (Supplementary Figures 4A–D). The result showed that the intra-group correlation was highest at *k* = 2, while the inter-group correlation was low, and could separate the samples in the TCGA data set relatively stable, which indicated that BC patients could be well-divided to two clusters (Supplementary Figure 4E). We also showed the classification of each sample (column) under the different number of clusters (*k*). When *k* = 2, most samples were divided into light blue and dark blue two parts (Supplementary Figure 4F). Therefore, we divided the training set of BC patients into cluster 1 (C1) and cluster 2 (C2). Next, the differential gene transcription profile and clinicopathological characteristics analysis were both displayed in the heatmap, which indicated that there was a significant separation between C1 and C2 of pyroptosis-related molecules. At the same time, we found that the clinicopathological characteristics were significantly correlated with differential genes related to pyroptosis. Among them, the expression level of Human Epidermal Growth Factor Receptor 2 (Her-2) might be different in the two groups, which needed to be further verified (Figure 2A). In the prognostic analysis, the OS and DFS of the two groups were markedly different. Cluster 2 presented a potentially better prognosis, but the difference was not significant in the field of statistics (Figures 2B,C). Furthermore, t-distributed stochastic neighbor embedding (t-SNE) dimensionality reduction analysis showed that the cluster was divided into two discrete clusters, C1 and C2 had different transcriptome characteristics (Figure 2D). These results indicated that pyroptosis might be correlated with the malignant progression and clinicopathological characteristics of BC.

### Functional Annotation of Different Subgroups

**Figure 3:**
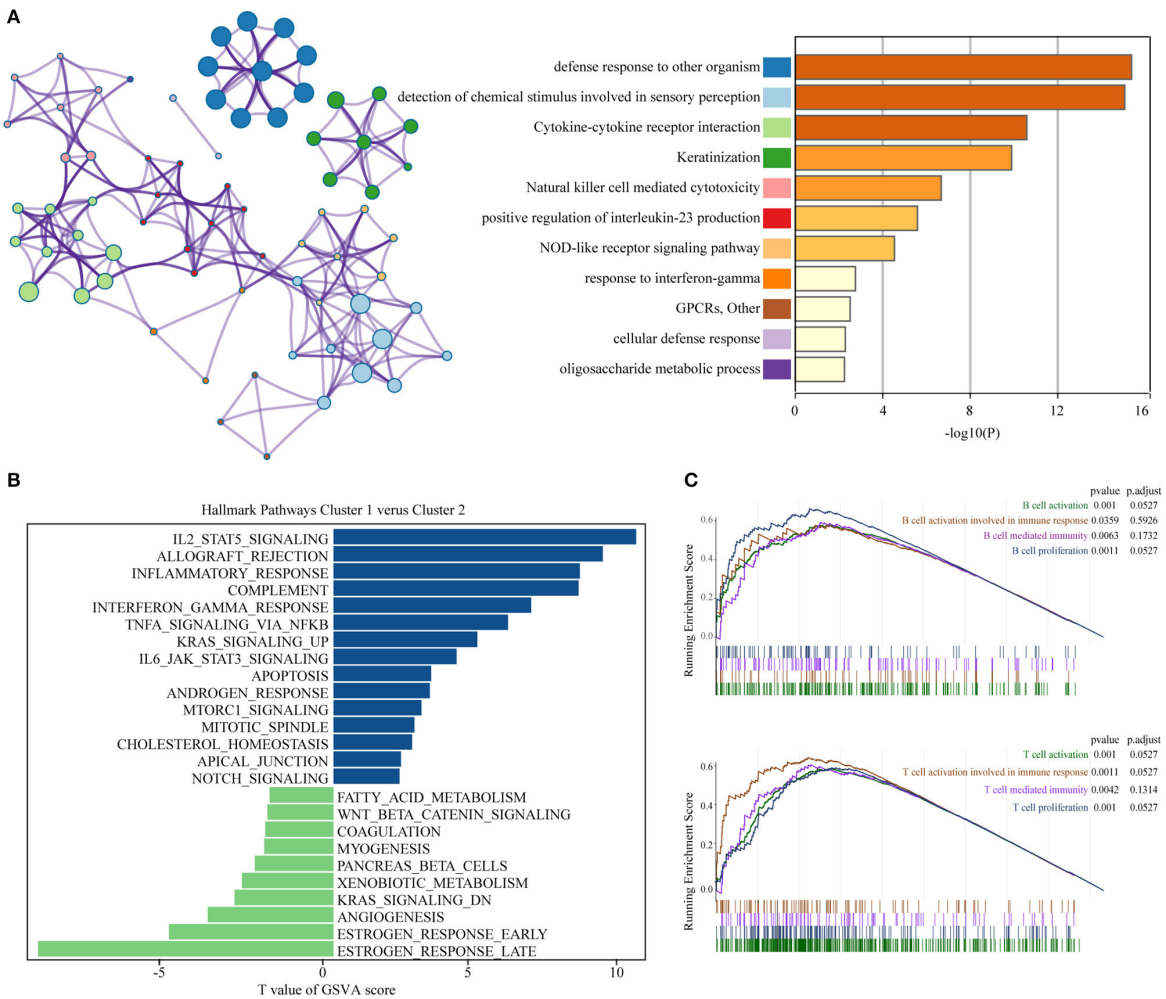
Functional annotation of different subgroups. **(A)** Significantly enriched top 20 pathways and interactions. **(B)** The GSVA between pyroptosis-related genes clusters1 and clusters2 using hallmark gene sets. **(C)** B cell and T cell related immune pathways are shown in GSEA. GSEA, Gene Set Enrichment Analysis; GSVA, Gene Set Variation Analysis.

To achieve exploring the different clinicopathological characteristics and prognosis of the two clusters of BC, we further investigated whether the genes screened by different subgroups had different biological processes. The top 200 most significant differentially expressed genes of pyroptosis-related genes clusters based on the *P*-value were selected to annotate the biological function. Metascape analysis of the upregulated genes revealed that “Cytokine-cytokine receptor interaction,” “Natural killer cell mediated cytotoxicity,” “positive regulation of interleukin-23 production,” “NOD-like receptor signaling pathway,” “response to interferon-gamma,” “cellular defense response,” and other inflammatory responses and immune-related pathways are significantly enriched in cluster 2, which might be closely related to the progression and distant metastasis of BC. The first 20 signally enriched bioinformatics pathways were listed in Figure 3A. We used GSVA to determine the degree of enrichment of different pathways between C1 and C2, the results showed that the inflammatory pathways such as “inflammatory response,” “interferon-γ response,” “TNF-α-NF-κB signaling,” “IL2-STAT5 signaling,” “IL6-JAK-STAT3 signaling” were significantly activated in C2 patients (Figure 3B). Then, we used GSEA to evaluate the activation of specific response pathways; and the analysis results pointed out that “B cell activation,” “B cell activation involved in immune response,” “B cell mediated immunity,” “B cell proliferation,” and the corresponding immune response of T cells were activated in C2 (Figure 3C). We explored the effects of pyroptosis-related genes on the prognosis. In general, the pyroptosis process promoted the activation of multiple inflammatory pathways, which might affect the tumorigenesis and progress of BC. In the cause of further exploring the specific effect of the pyroptosis process on the prognosis of BC, we further developed a prognostic model.

### The Prognostic Value of Pyroptosis-Related Genes and the Construction of Risk Score Model

**Figure 4:**
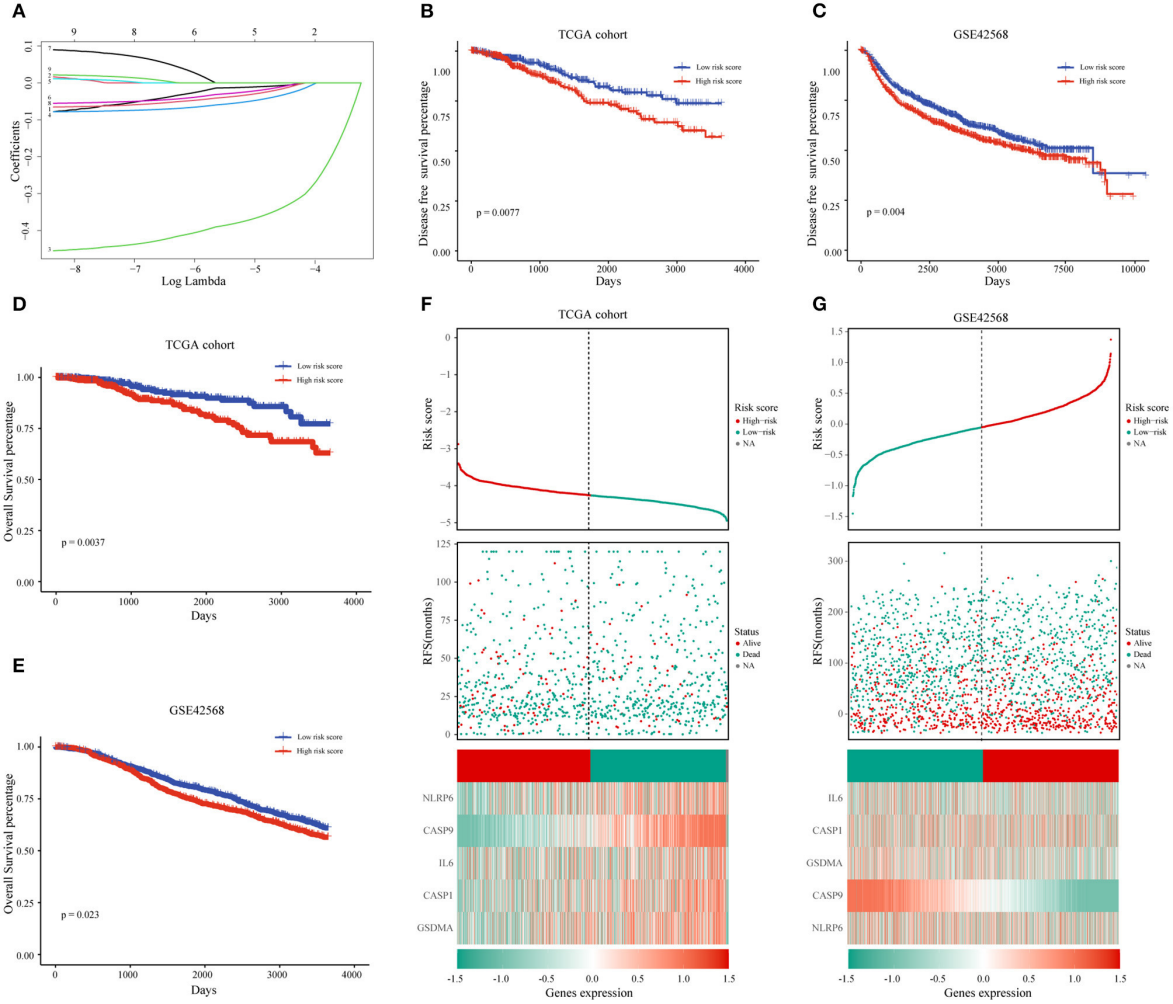
The prognostic value of pyroptosis-related genes and the construction of risk score model. **(A)** The coefficients of five pyroptosis-related genes in LASSO Cox regression were calculated. **(B,D)** Kaplan-Meier analysis of OS and DFS in patients with low- and high-risk scores in TCGA dataset. **(F)** BC patients with high-risk scores had a worse prognosis in the TCGA database. **(C,E,G)** The same analysis method is used to verify the risk score characteristics in the GSE42568 data set. TCGA, The Cancer Genome Atlas; DFS, Disease-Free Survival; OS, Overall Survival.

After we have known the relationship between pyrolysis-related genes and BC progression, we further clarified the influence of 33 pyroptosis-related genes on the prognosis of BC with the help of univariate analysis in the TCGA training dataset. A total of nine genes including IL-18, IL-6, CASP1, CASP4, CASP9, NLRP6, NLRP3, PYCARD, GSDMA were selected according to *P*-value < 0.2 for further analysis. The prognostic risk score features were constructed through the LASSO regression model based on the minimum criteria, including NLRP6, IL-6, CASP1, CASP9, and GSDMA (Figure 4A). By using the five genes screened and corresponding coefficients to construct a risk score feature, and the risk score formula was as follows: Risk score = (−0.0318 × the expression of NLRP6) + (−0.0499 × the expression of GSDMA) + (−0.0234 × the expression of IL-6) + (−0.0114 × the expression of CASP1) + (−0.3714 × the expression of CASP9), In our risk score, the five selected genes were tumor-suppressor genes.

To learn more about the prognostic value of the risk score, BC patients were divided into high-risk group and low-risk group based on the median of the risk score in the TCGA database. The results indicated that the high-risk score group was associated with a higher mortality rate (Figure 4F). Survival analysis based on OS and DFS clarified that high-risk score group had a worse prognosis than those with low-risk scores (Figures 4B,D). At the same time, we named the GSE42568 dataset as the validation set to achieve the aim of verifying the prediction power of the risk score, and the results of the validation set and the training set were the same (Figures 4C,E,G).

### The Relationship Between the Risk Score and Clinical Characters in Breast Cancer Patients

**Figure 5:**
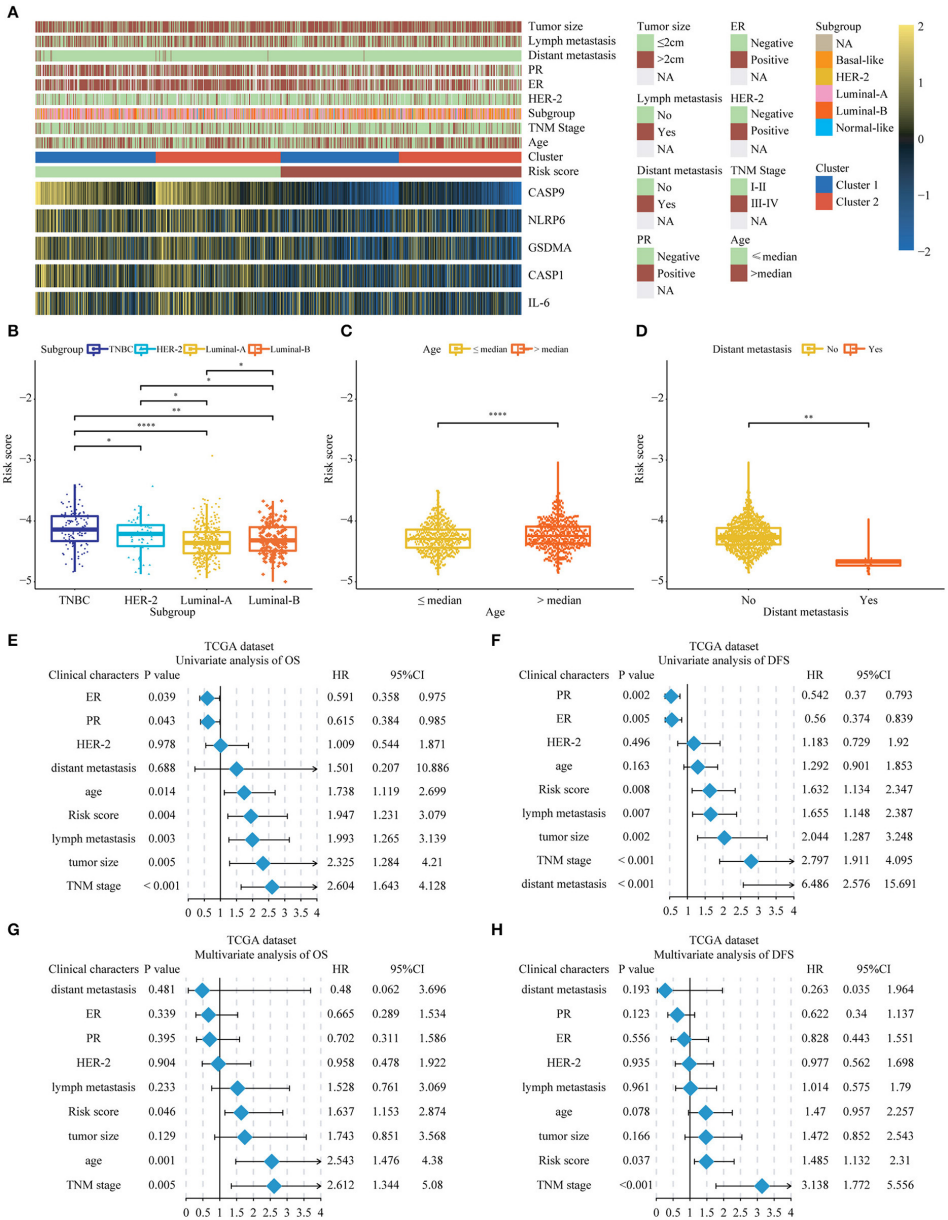
The relationship between the risk score and clinical characters in Breast cancer patients. **(A)** The relationship between risk score, cluster 1, cluster 2, and 5 key genes and clinical case characteristics of BC patients. **(B–D)** The relationship between risk score and clinical characteristics, including PAM50 classification, age and distant metastasis. **(E–H)** Through univariate and multivariate analysis, the risk score was an independent risk factor for the prognosis of breast cancer in the TCGA database. *P < 0.05, **P < 0.01, ****P < 0.0001. TNM, Tumor Node Metastasis; DFS, Disease-Free Survival; OS, Overall Survival; TNBC, triple-negative breast cancer; HER-2, Human Epidermal Growth Factor Receptor 2; PR, progesterone receptor; ER, estrogen receptor; TCGA, The Cancer Genome Atlas.

Next, we further researched on the correlation among the pyroptosis-related genes and risk scores and the clinical features of BC. The heat map represented the expression and clinical characteristics of pyroptosis-related genes in high- and low-risk patients (Figure 5A; Supplementary Tables 2, 3). We found that age, TNM stage and distant metastasis had a close relationship with the high-risk score. Subsequently, we assessed the difference between the risk score and each clinical feature. It could be seen from the histogram that triple-negative breast cancer (TNBC) had the highest risk score, the highest risk of disease progression and recurrence, and the worst prognosis. The results of this analysis were consistent with previous studies of TNBC. The results of the other three subgroups Her-2, Luminal-A, and Luminal-B were also consistent with the National Comprehensive Cancer Network (NCCN) guidelines. The risk score we designed could help determine the clinicopathological classification of BC. If the risk score was higher, the clinical classification was closer to the TNBC or Her-2 subtype. In addition, we found that a higher risk score meant a worse prognosis (Supplementary Figure 5). Conversely, the lower the patient's score, the more likely the classification result was Luminal-A or Luminal-B subtype (Figure 5B). We found age and lymphatic metastasis were important factors affecting risk scores (Figures 5C,D). Then, univariate and multivariate Cox regression analyses were conducted on the TCGA dataset. We observed that estrogen receptor (ER), progesterone receptor (PR), tumor size, age, lymph metastasis, TNM stage, and distant metastasis were significantly correlated with prognosis by the univariate analysis (Figures 5E,F). Then, the risk score remained strongly associated with the DFS by multivariate analysis (Figures 5G,H). The above showed that the risk score established by pyroptosis molecules was an independent predictor of the prognosis and progression of BC.

### The Expression Level of Five Key Pyroptosis-Related Genes in Breast Cancer Patients

**Figure 6:**
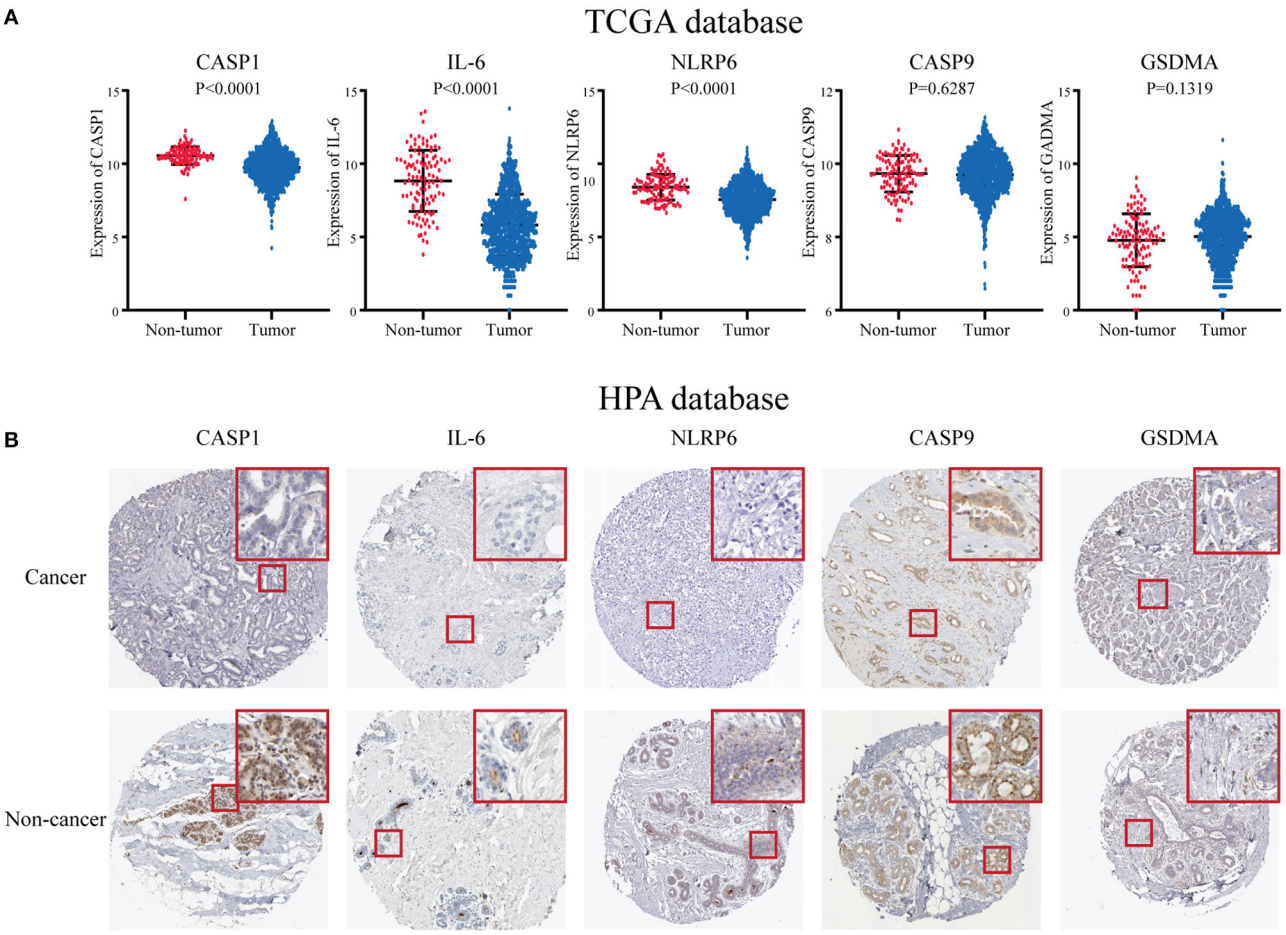
The expression level of five key pyroptosis-related genes in breast cancer patients. **(A)** The expression of five key genes is in the TCGA database. **(B)** The expression of five key genes in the HPA platform. HPA, Human Protein Atlas; TCGA, The Cancer Genome Atlas.

To further research the five genes included in the risk score, we identified these in the TCGA database, and the results showed that three genes including CASP1, IL-6, and NLRP6 were significantly decreased in the BC patients at the RNA level (Figure 6A). While there was no significant difference in CASP9 and GSDMA between BC and paracarcinoma tissues. Then, we used the HPA database to evaluate the expression difference at the protein level. The results indicated that the protein levels of NLRP6, IL-6, CASP1 in BC tissues were significantly decreased vs. normal tissues (Figure 6B). The expression level of CASP9 and GSDMA in BC and paracarcinoma tissues was similar. The results at the protein level were consistent with the RNA expression data we found earlier.

## Discussion

Although improvement has happened in therapeutic effects and prognosis these years, progression and metastasis are still the main causes of death in BC patients (17). Existing treatment and testing methods can not cover all BC patients, and carcinogenesis, malignant progression, and recurrence involve complex multistep processes and still to be fully elucidated. Thus, it is crucial to understand the potential in review mechanisms involved, to develop powerful prognostic predictors, and explore novel therapeutic targets. After clarifying the differences in the expression of pyroptosis-related genes in BC and paracarcinoma tissues, in order to quantify the impact of pyrosis on the progression, a risk score was constructed to evaluate the prognosis of patients. At the same time, a part of the possible mechanism was discussed.

In this study, we firstly reported the expression levels of 33 pyroptosis-related genes in BC and paracarcinoma tissues and identified that they were differentially expressed. The analysis results showed that the expression of five molecules such as CASP3, CASP6, GSDMD, NOD2, PYCARD in BC tissues was higher than that in adjacent tissues. Then, we found that somatic mutations of pyroptosis-related factors were more common in BC. Many previous studies have confirmed that pyroptosis-related genes had a close relationship with the tumorigenesis and progression in many cancers. Interleukin-6-mediated activation of STAT3 in fibroblasts played a key role in driving colorectal tumors (18). However, there was no significant difference in clinical characteristics between the two clusters which were figured out through the consensus clustering analysis. In order to further evaluate the prognostic value of these pyroptosis-related genes, a five-genes risk signature was constructed based on univariate analysis and LASSO Cox regression analysis in the TCGA database, and we validated it in a GEO dataset with good performance. And the DEGs between the high- and low-risk group had a close relationship with immune-related and inflammatory response pathways by the functional analysis. Comparing the degree of pathway enrichment between the low-risk group and the high-risk group, we found that the high-risk group reduced specific immunity and non-specific immunity-related pathway activities versus the low-risk group.

The risk score we constructed contains five pyroptosis-related molecules including IL-6, CASP1, CASP9, NLRP6, GSDMA, etc. Interleukin-6 influenced the proliferation, differentiation and anti-apoptosis of common malignant tumor cells. Overexpression of IL-6 and its receptors were usually found in BC, prostate cancer, and oral squamous cell carcinoma. Interleukin-6 could activate the potential autocrine/paracrine Notch-3/Jagged-1 ring to improve the self-renewal of breast stem cells (19). Interleukin-6 and its downstream effector STAT3 constituted a key carcinogenic pathway (20), so targeting IL-6 may bring benefits to BC patients. Previous studies confirmed that the apoptosis caused by CASP1 involves the Bid-caspase-9-caspase-3 axis, which could lead to pyroptosis mediated by GSDME. Caspase-1 is involved in pyroptosis. The effect was clearer than in apoptosis (21). Recently, it was discovered that caspase-1 mediated GSDMD proteolysis, the formation of GSDMD membrane pores, cell lysis, and pyroptosis. Gasdermin D was a substrate of caspase-11 and CASP1 and mediates cell death (22). Many pro-apoptotic signals could activate caspase-9, which is an initiating protease. It was an initiator and does not need to be cleaved, but only needed to be activated to activate caspase-3 and downstream caspase to initiate cell death. Caspase-9 had an important effect on the apoptosis of a variety of cancer cell types, and its positive and negative regulators were reported in the previous literature (23). The expression of GSDMA was significantly increased in the skin and gut, however, it was depleted in gastric cancer (24). But GSDMA polymorphism was associated with childhood asthma, inflammatory bowel disease and systemic sclerosis (25). NLR pyrin domain-containing protein 6 (originally named PYPAF5) belonged to the NOD-like receptor family, and together with NLRP4, NLRP7, NLRP3, and NLRC1 constituted the ability to construct a fully operational inflammasome (26). The inflammasome NLRP6 played a vital part in adjusting inflammation and hosted resistance to gut microbiomes. Water shortage stress could cause NLRP6 inhibition and alterations in the composition of the gut microbiome, and mice were more likely to suffer from intestinal inflammation (27). Colitis and colorectal tumors were significantly increased in mice lacking NLRP6 (28). The cytosolic lipoteichoic acid bound to and activated NLRP6, which aggravated systemic Gram-positive pathogen infection by producing IL-18, which was a new innate immune pathway (29). In this experiment, we found that the expression of NLRP6 was different in BC and paracarcinoma tissues, with low expression in BC tissues and relatively high expression in paracarcinoma tissues. The coefficient was negative when the risk score model was subsequently established, which was consistent with the previous experimental results.

This study also had many limitations. Firstly, this was a retrospective analysis which cannot draw a causal relationship between abnormal gene expression and BC, so future prospective research is needed. In addition, additional *in vitro* and *in vivo* functional analyses were required to validate and extend these results. The results obtained by pure bioinformatics analysis can only be used to predict conclusions and are not sufficient for obtaining accurate conclusions. Finally, we only explored the possible mechanism of pyroptosis in BC at the level of bioinformatics, and the specific mechanisms of related molecules in BC were still to be studied.

## Conclusion

Our research confirmed that the expression of pyroptosis-related genes was different in BC and normal breast tissues and clarified the close relationship between changes in the expression of pyroptosis-related genes and the malignant progression of BC. The characteristics of pyroptosis-related genes were identified and verified, which could accurately predict the prognosis of BC patients. These results provided evidence for the biomarkers of BC to judge the progress and prognosis and provided a new potential therapeutic target for targeted therapy. In the future, editing of genes related to pyrolysis may become an effective option for BC treatment through gene therapy.

## Data Availability Statement

Publicly available datasets were analyzed in this study. This data can be found here: The datasets generated for this study can be found in TCGA (*n* = 1,222, 1,109 BC samples and 113 normal breast samples) and separate GEO microarray datasets (GSE42568, 104 BC samples and 17 normal breast samples).

## Ethics Statement

Ethical review and approval was not required for the study on human participants in accordance with the local legislation and institutional requirements. Written informed consent for participation was not required for this study in accordance with the national legislation and the institutional requirements.

## Author Contributions

ZL and XG designed the study and revised the manuscript. TR and JZ analyzed the data and wrote the manuscript. All authors contributed to the article and approved the submitted version.

## Conflict of Interest

The authors declare that the research was conducted in the absence of any commercial or financial relationships that could be construed as a potential conflict of interest. The reviewer ZR and RS declared a shared parent affiliation, with no collaboration, with the authors to the handling editor at the time of the review.

## Publisher's Note

All claims expressed in this article are solely those of the authors and do not necessarily represent those of their affiliated organizations, or those of the publisher, the editors and the reviewers. Any product that may be evaluated in this article, or claim that may be made by its manufacturer, is not guaranteed or endorsed by the publisher.
