# Supplementary figures and images for: Pyroptosis-Related Signatures for Predicting Prognosis in Breast Cancer
Source: Front Surg. 2022 Feb 8;9:788437. doi: 10.3389/fsurg.2022.788437 (PMC8861281; doi:10.3389/fsurg.2022.788437)

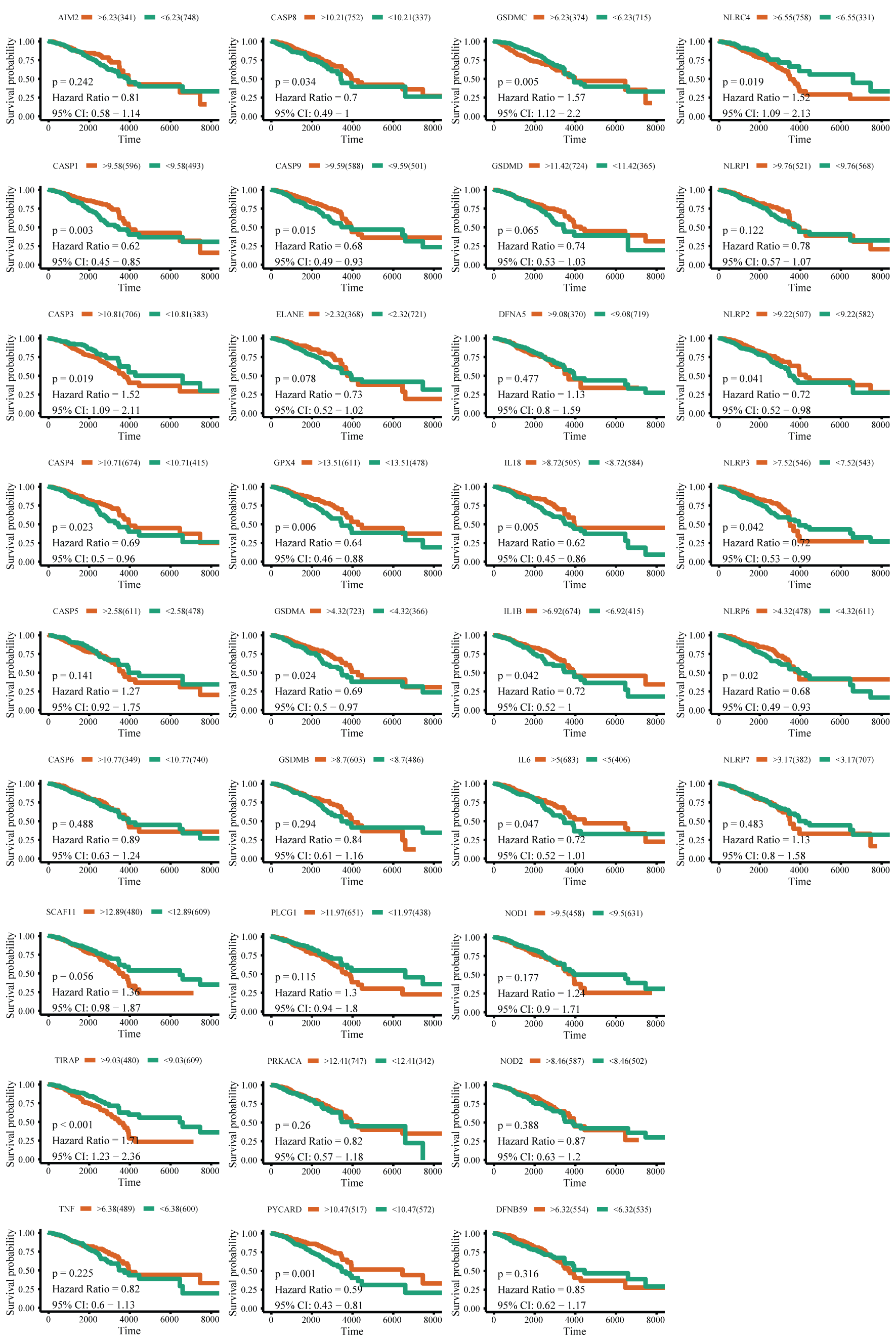

Supplement: Supplementary file 2 [file Image_1.TIF]

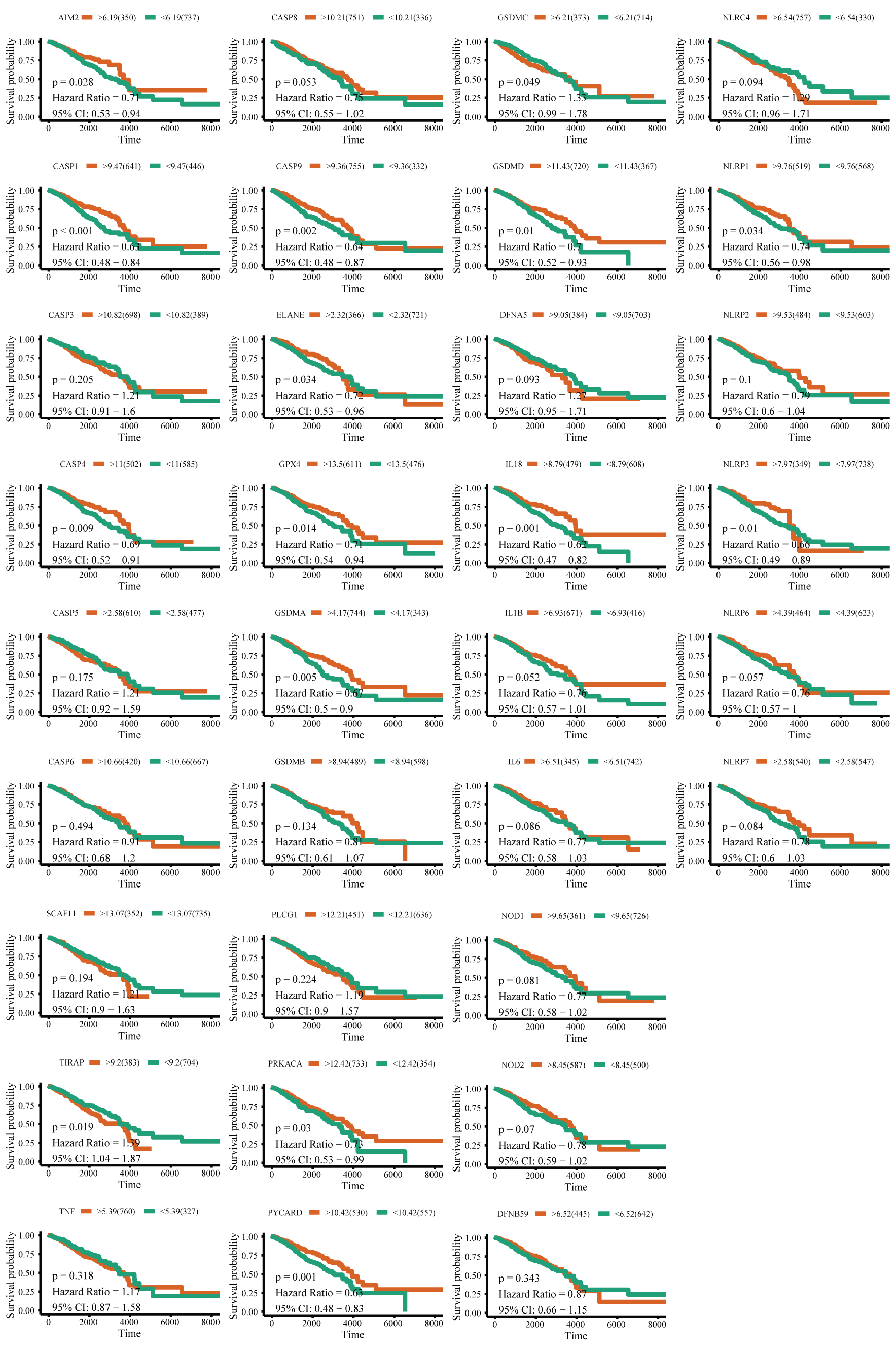

Supplement: Supplementary file 3 [file Image_2.TIF]

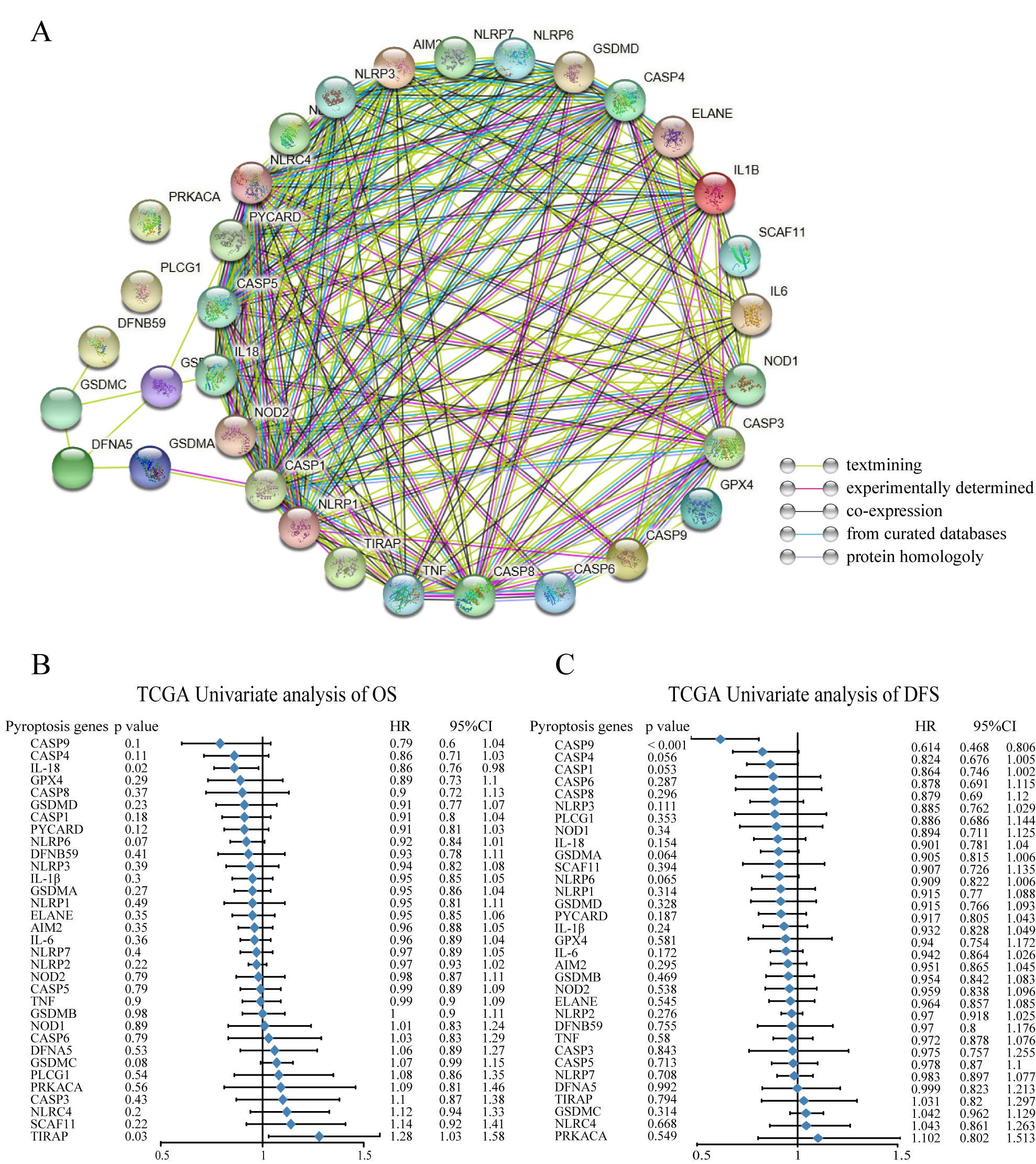

Supplement: Supplementary file 4 [file Image_3.TIF]

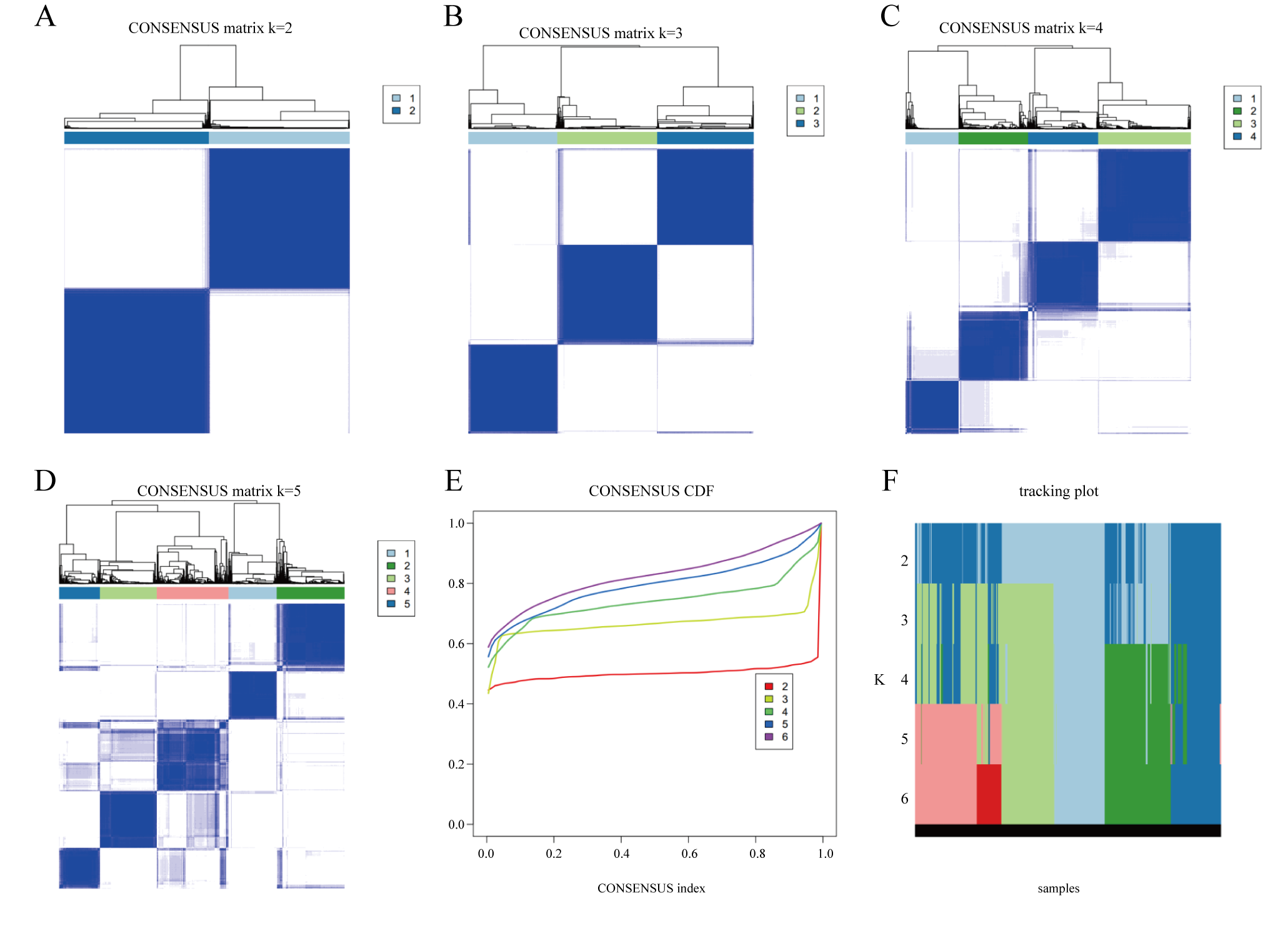

Supplement: Supplementary file 5 [file Image_4.TIF]

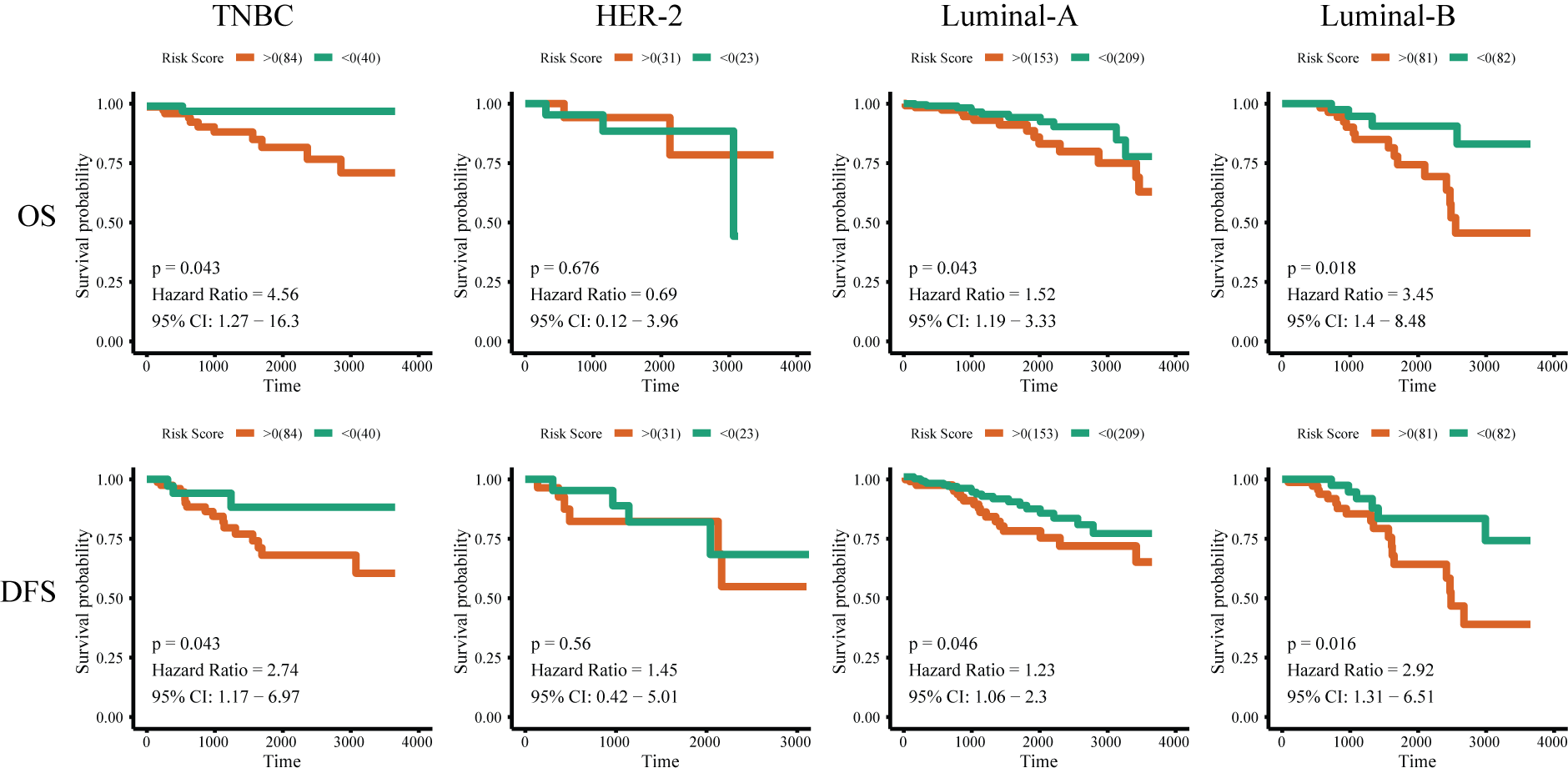

Supplement: Supplementary file 6 [file Image_5.TIF]
